# Comment on Chandler et al. 23-Valent Pneumococcal Polysaccharide Vaccination Does Not Prevent Community-Acquired Pneumonia Hospitalizations Due to Vaccine-Type *Streptococcus pneumoniae*. *Microorganisms* 2022, *10*, 560

**DOI:** 10.3390/microorganisms10101987

**Published:** 2022-10-08

**Authors:** Nicole Cossrow, Rennie Joshi, Kenneth Klinker, Ulrike K. Buchwald

**Affiliations:** Merck & Co., Inc., Rahway, NJ 07065, USA

**Keywords:** pneumococcal conjugate vaccines, pneumococcal disease, pneumococcal serotypes, community-acquired pneumonia, test-negative design

## Abstract

The 23-valent pneumococcal polysaccharide vaccine (PPSV23) targets 23 common serotypes and is recommended for use in adults in various countries to protect against pneumococcal infection. Test-negative design (TND) studies aim to include cases and controls from the same healthcare facilities; however, design choices or limitations associated with conducting real-world research can affect the study results. Here, we highlight how some methodological limitations may have affected results and conclusions of a published study described by Chandler et al.

In an article published in March 2022, Chandler et al. reported their assessment of the effectiveness of 23-valent pneumococcal polysaccharide vaccine (PPSV23) for the prevention of community-acquired pneumonia (CAP) hospitalizations due to vaccine serotypes in adults in Louisville, Kentucky, between June 2014 and March 2017 [1]. The study used a test-negative design (TND) to estimate vaccine-type (VT) vaccine effectiveness (VE) of PPSV23. The authors reported the crude and adjusted VEs in this study to be 17% (95% confidence interval [CI]: −13% to 40%) and 14% (95% CI: −17% to 38%), respectively, and concluded that PPSV23 is not effective in preventing PPSV23 VT-CAP hospitalizations. As with any study, it is important to consider how study design choices or limitations associated with conducting real-world research can affect the study results. Below, we highlight how some methodological limitations likely affected the results and conclusions of the study described by Chandler et al.

First, in this study by Chandler et al., individuals who received a PPSV23 vaccination within 30 days prior to, or more than 5 years prior to, their CAP hospitalization were considered non-vaccinated. This may underestimate the true effectiveness of PPSV23 and may bias the VE estimate towards the null hypothesis. While some evidence suggests that immunity following PPSV23 vaccination wanes after 5 years, there are studies that show immunity exists after 5 years [2,3]. Including these individuals in the control group instead of the vaccinated group may create differential unidirectional misclassification bias. Although a sensitivity analysis that excluded 61 individuals who received a PPSV23 dose 30 days prior to, or more than 5 years prior to, their CAP admission showed unadjusted effectiveness of PPSV23 to be 17% (95% CI: −13% to 40%), an analysis was not performed in which these individuals were included in the vaccinated group, rather than excluded entirely. Even a small change in allocation group status would lead to a substantial change in VE. For example, moving approximately 1.5% (*n* = 50) or 3% (*n* = 100) of the controls from the unvaccinated to the vaccinated group changes the crude VE from 17% to 25% or 32%, respectively (Table 1).

Second, Chandler et al. chose to exclude 463 individuals who received 13-valent pneumococcal conjugate vaccine (PCV13) from the study. We believe this was done because the focus of the study was to investigate whether PPSV23 could prevent hospitalized VT-CAP. However, by excluding individuals (both cases and controls) who received PCV13, many individuals who may have also received PPSV23 were, in turn, excluded. Since most participants were controls, the majority of the 463 excluded participants would also be controls. Furthermore, PCV13 was first recommended in the United States in 2014, and the guidance was for PCV13 to be followed by PPSV23 [4]. Therefore, the excluded individuals may have received both PCV13 and PPSV23 and consequently were more likely to be controls than cases. For these reasons, the VE is likely biased toward the null. Notably, in a TND study by McLaughlin et al. that evaluated the VE of PCV13 against hospitalized VT-CAP in older adults in Louisville, Kentucky, the researchers did not exclude individuals vaccinated with PPSV23 from the study, thus not introducing potential bias [5].

Third, a limitation that is especially relevant to real-world evidence studies carried out in the United States relates to the fact that vaccination records from US electronic medical records (EMRs) or primary insurers are not always reliable. In the study by Chandler et al., individuals were excluded if vaccination status was not documented in an individual’s EMR or primary insurer record. Among the PPSV23-vaccinated individuals, EMRs were the primary source of vaccination status in 87% of individuals, with the remainder by the primary insurer (13%). Additional limitations may be associated with the use of EMR sources, including a lack of connection from different providers and under-reporting [6,7]. If interoperability of EMRs in the region was not implemented, there is risk of misclassification bias through EMR if an adult immunization registry is unavailable [8]. Indeed, in their study evaluating the VE of PCV13, McLaughlin et al. seem to have acknowledged this limitation by sourcing data solely from insurers [5]. However, switching insurers is common at or around 65 years of age in the United States when individuals become eligible for Medicare. This switching of insurers can result in a loss of vaccination records, which, in turn, may have led to an underestimate of the true PPSV23 vaccine exposure status in this population and bias VE toward the null. Furthermore, most individuals included in the study by Chandler et al. were controls, with a median age of 66 years versus 62 years for cases, hence leading to a potential underestimate of the true PPSV23 vaccine exposure in the control group.

TND studies aim to include cases and controls from the same healthcare facilities, thereby accounting for similar participation rates, information quality and completeness, diagnostic suspicion tendencies, and preferences by doctors [9]. In addition, TND studies are designed to reduce bias associated with confounding by healthcare-seeking behaviour and misclassification; however, this is not always possible [10] and may have been difficult to capture because of the median age differences in the Medicare-eligible individuals ≥65 years of age. Furthermore, exclusion of individuals due to missing data indicates that the source population may not be appropriately representative, and this may undermine the generalizability of the results. Indeed, more than half of the eligible participants did not participate because they did not provide urine samples or proof of vaccination status; those who chose to provide a urine sample may be very different by exposure/case–control status. Furthermore, the large exclusion of participants indicates that the source population may not be appropriately represented and calls into question the generalizability of the results. In addition, covariate selection, whether data driven or via a priori selection, can affect the results. Not adjusting for confounders (when there is evidence of confounding or known confounders) can also affect the point estimate.

Lastly, the likelihood of selection bias must be addressed when examining the differing baseline characteristics between the PPSV23-vaccinated and unvaccinated cohorts. For example, individuals in the vaccinated group were significantly older (median age: 68 versus 65 years of age; *p* = 0.001) and more likely to have comorbidities including diabetes, chronic obstructive pulmonary disease, renal disease, hypertension, hyperlipidemia, and heart disease. These differences in key participant characteristics and the absence of age-matched controls represent confounders. By contrast, the PCV13 TND study conducted by McLaughlin and colleagues was limited to people ≥65 years of age in both cases and controls [5]. It is noteworthy that McLaughlin et al. also adjusted for self-reported influenza vaccination as a possible confounder, whereas Chandler et al. did not [5]. Similarly, Chandler et al. did not adjust for sex and race, and these variables are generally considered confounders, while potential other residual confounders, such as smoking and influenza status, should also have been adjusted for in the multivariate analyses. It would be helpful to understand why the study design approach differed between the PCV13 study [5] and the PPSV23 study [1]; for example, the PCV13 TND study conducted sensitivity analyses for two different control groups. In addition, no sensitivity analysis was conducted for the propensity to be vaccinated, which was recently highlighted as an important factor in a coronavirus disease 2019 (COVID-19) TND study [11,12].

Measuring accurate VE against VT-CAP can be challenging, particularly as identifying CAP can be difficult; for example in the case of non-hospitalized patients [13]. Therefore, we recognize the efforts exhibited by the authors to test the effectiveness of PPSV23 for the prevention of hospitalized VT-CAP; however, taking into account our aforementioned concerns regarding the study design and analysis, we believe the overall conclusions as stated by Chandler et al. in the article should be interpreted with some degree of caution.

## Figures and Tables

**Table 1:** Effect on vaccine effectiveness if 1.5% or 3% of the control population were considered vaccinated.

	Base Case		1.5% of Controls Considered Vaccinated		3% of Controls Considered Vaccinated	
	*VT CAP Case*	*Non-VT* *CAP Control*	*VT CAP Case*	*Non-VT* *CAP Control*	*VT CAP Case*	*Non-VT* *CAP Control*
**PPSV23** **vaccinated**	48	560	48	610	48	660
**Not vaccinated**	288	2790	288	2740	288	2690
**OR**	**0.83** (48 × 2790)/(288 × 560)		**0.75** (48 × 2740)/(288 × 610)		**0.68** (48 × 2690)/(288 × 660)	
**VE**	**17%** (1−0.83) × 100%		**25%** (1−0.75) × 100%		**32%** (1−0.68) × 100%	

OR = odds ratio; PPSV23 = 23-valent pneumococcal polysaccharide vaccine; VE = vaccine effectiveness; VT-CAP = virus-type community-acquired pneumonia.
